# High-level *Plasmodium falciparum* sulfadoxine-pyrimethamine resistance with the concomitant occurrence of septuple haplotype in Tanzania

**DOI:** 10.1186/s12936-015-0977-8

**Published:** 2015-11-05

**Authors:** Vito Baraka, Deus S. Ishengoma, Filbert Fransis, Daniel T. R. Minja, Rashid A. Madebe, Deogratius Ngatunga, Jean-Pierre Van Geertruyden

**Affiliations:** National Institute for Medical Research, Tanga Research Centre, P. O. Box 5004, Tanga, United Republic of Tanzania; International Health Unit, Department of Epidemiology, University of Antwerp, Campus Drie Eiken, Universiteitsplein 1, 2610 Wilrijk, Belgium; Tanzania Food and Drugs Authority, P: O. Box 77150, Dar es Salaam, United Republic of Tanzania

**Keywords:** *Plasmodium falciparum*, Single nucleotide polymorphisms, Sulphadoxine-pyrimethamine, Molecular markers, Septuple *Pfdhfr*, *Pfdhps*, Tanzania

## Abstract

**Background:**

Tanzania abandoned sulfadoxine-pyrimethamine (SP) as the first-line treatment for uncomplicated malaria in 2006 due to high levels *Plasmodium falciparum* resistance. However, SP is still being used for intermittent preventive treatment during pregnancy (IPTp-SP). This study aimed to assess the pattern of *P. falciparumdihydrofolate reductase(Pfdhfr)* and *dihydropteroate synthetase (Pfdhps)* mutations and associated haplotypes in areas with different malaria transmission intensities in mainland Tanzania, 6 years after withdrawal of SP as a first-line treatment regimen for uncomplicated malaria.

**Methods:**

A total of 264 samples were collected during cross-sectional surveys in three districts of Muheza, Muleba and Nachingwea in Tanga, Kagera and Lindi regions, respectively. Parasite genomic DNA was extracted from *P. falciparum* positive samples. The *Pfdhfr, Pfdhps* single nucleotide polymorphisms (SNPs) were amplified using nested polymerase chain reaction and detected by sequence specific oligonucleotide probe-enzyme linked immunosorbent assay (SSOP-ELISA).

**Results:**

The prevalence of the mutant *Pfdhfr*-*Pfdhps* haplotypes was heterogenous and transmission dependent. The triple *Pfdhfr* mutant haplotypes (C**IRN**I) were predominant in all sites with significantly higher frequencies at Muheza (93.3 %) compared to Muleba (75.0 %) and Nachingwea districts (70.6 %), (p < 0.001). Overall, the prevalence of the wild-type *Pfdhps* (SAKAA) haplotype was lowest at Muheza (1.3 %), (p = 0.002). Double *Pfdhps* haplotype S**GE**AA was significantly high at Muheza (27.2 %) and Muleba (20.8 %) while none (0 %) was detected at Nachingwea (p < 0.001). The prevalence of triple *Pfdhps* S**GEG**A haplotype was significantly higher at Muheza compared to Muleba and Nachingwea (p < 0.001). In contrast, Nachingwea and Muleba had significantly higher prevalence of another triple *Pfdhps***AGE**AA haplotype (χ^2^ = 39.9, p < 0.001). Conversely, *Pfdhfr*-*Pfdhps* as quintuple and sextuple haplotypes were predominant including the emergence of a septuple mutant haplotype C**IRN**I-**AGEG**A (n = 11) observed at Muheza and Muleba.

**Conclusion:**

These results ascertain the high prevalence and saturation of *Pfdhfr* and *Pfdhps* haplotypes conferring SP resistance in areas with changing malaria epidemiology; and this could undermine the use of IPTp-SP in improving pregnancy outcomes. In these settings where high level SP resistance is documented, additional control efforts are needed and evaluation of an alternative drug for IPTp is an urgent priority.

## Background

*Plasmodium falciparum* malaria remains a major public health problem in the sub-Saharan Africa (SSA). Increased global efforts in malaria control and elimination have resulted into significant reduction in the disease burden through scaling up of control interventions such as use of insecticide-treated nets (ITNs), indoor residual spraying (IRS) and early case diagnosis and prompt treatment using effective anti-malarial drugs [1]. However, malaria control programmes are repeatedly challenged by rapid and widespread anti-malarial drug resistance [2–4]. Following the widespread drug resistance to sulfadoxine-pyrimethamine (SP) and chloroquine (CQ) [5, 6], the World Health Organization (WHO) recommended a policy change from monotherapy to artemisinin-based combination therapy (ACT) [7]. Despite policy changes as a result of widespread drug resistance against SP, the drug is still being recommended for intermittent preventive treatment during pregnancy (IPTp-SP) whereby in areas of moderate to high transmission SP dose is given at each scheduled antenatal care (ANC) visit at least monthly to prevent pregnancy associated malaria (PAM) and improve pregnancy outcomes [8]. In addition, SP is still being used in the IPT in infants (IPTi-SP) to reduce malaria and anaemia among infants as well as seasonal malaria chemoprevention (SMC) programmes in some malaria endemic settings [9]. Nonetheless, the chemoprophylactic effectiveness of IPTp-SP, IPTi-SP and SMC-SP strategies against malaria control amongst the most vulnerable is increasingly being compromised due to the rapid and widespread SP-resistance.

In Tanzania, SP was introduced as the first-line anti-malarial drug for treatment of uncomplicated falciparum malaria in 2001 as a result of high level chloroquine (CQ) resistance (CQR) and clinical treatment failures (TFs) [10]. However, 5 years after its introduction, the policy was again revised in November 2006 due to widespread resistance against SP and remarkable TFs [4, 11]. Thus, artemether-lumefantrine (AL), an ACT, was introduced as the first-line treatment for uncomplicated *falciparum* malaria in the mainland Tanzania [12]. The changes in malaria treatment policies were supported by data from molecular epidemiological resistance surveillance against CQ and SP. The in vivo molecular surveillance ascertained the treatment failures (TFs) [4, 11] with *P. falciparum* resistance against CQ and SP in clinical settings.

Sulfadoxine–pyrimethamine acts by inhibiting the *P. falciparum* dihydrofolate reductase (DHFR) and dihydropteroate synthetase (DHPS) enzymes [13, 14]. Notably, several single nucleotide polymorphisms (SNPs) in the *Pfdhps* gene at codons Serine 436 to Alanine (S436A), Alanine 437 to Glycine (A437G), Lysine 540 Glutamic acid (K540E), Alanine 581 to Glycine (A581G) and Alanine 613 to Serine (A613S) are associated with sulfadoxine resistance. Pyrimethamine resistance is conferred by mutations in the *Pfdhfr* gene resulting from amino acid substitution at codons Cysteine 50 to Arginine (C50R), Asparagine 51 to Isoleucine (N51I), Cysteine 59 to Arginine (C59R), Serine 108 to Asparagine/Threonine (S108 N/T), and Isoleucine 164 to Leucine (I164L) [15, 16]. Emergence and subsequent accumulation of the mutations in both the *Pfdhfr/Pfdhps* genes is associated with SP clinical TF in several epidemiological settings [11, 17–19]. The major *Pfdhfr* haplotypes emerge as a result of combination of mutations of the wildtype cysteine-asparagine-cysteine-asparagine-isoleucine (CNCNI) followed by the gradual changes resulting to cysteine-isoleucine-cysteine-**asparagine**-isoleucine (CIC**N**I), cysteine-asparagine-**arginine-asparagine**-isoleucine (CN**RN**I), cysteine-isoleucine-**arginine-asparagine**-isoleucine (C**IRN**I) and cysteine-**isoleucine-arginine-asparagine-leucine** (C**IRNL)** as a single, double, triple and quadruple mutants, respectively (at amino acid positions C50**R**, N51**I**, C59**R**, S108**N**, and L164**I**) [20, 21]. For *Pfdhps*, the wild type genotype serine-alanine-lysine-alanine-alanine (SAKAA) can change to a single **alanine**–alanine-lysine-alanine-alanine or serine-**glycine**-lysine- alanine–alanine (**A**AKAA or S**G**KAA), double (**AG**KAA, **SG**K**G**A or S**GE**AA) or triple mutants (**AGE**AA or S**GEG**A, at amino acid positions S436**A**, A437**G**, K540**E**, A581**G** and A613**S/T**) [14, 22, 23]. Quintuple mutations as a result of the combination of *Pfdhfr* triple (C**IRN**I) and the *Pfdhps* double (A437**G**, K540**E**) mutations were reported in East Africa [24, 25] and have been shown to confer high level SP resistance rendering SP ineffective for treatment of *P. falciparum* infections. In northeastern Tanzania, the rise in *Pfdhps* mutations at codons A581**G** has led to higher proportions of S**GEG**A haplotypes [26]. Combined *Pfdhfr*-*Pfdhps* S**GEG**A-C**IRN**I forming the sextuple haplotype has been associated with sub-optimal prophylactic effect and thus poor pregnancy outcomes particularly in areas where SP resistance is widespread in sub-Saharan Africa [17, 18, 27].

In fact, WHO recommends stopping IPTp-SP in areas where the *Pfdhfr* K540**E** prevalence is >95 % and *Pfdhps* A581G is >10 % as SP is likely to be ineffective [28]. The widely spread highly resistant haplotypes including quintuple (C**IRN**I-S**GE**AA) and sextuple (C**IRN**I-S**GEG**A) are likely to compromise effectiveness of IPTp-SP strategy. Continuous surveillance of SP effectiveness using molecular markers is therefore, critical and should be routinely implemented as recommended by WHO [29].

Determination of molecular markers in the *Pfdhfr* and *Pfdhps* genes offers an invaluable tool for epidemiological surveillance of SP resistance in malaria endemic settings and will generate important data to assist and inform malaria control programmes on the status of resistance particularly due to emergence and rapid spread of highly resistant mutations and haplotypes that may highly compromise the usefulness of IPT strategies. This study aimed to assess the status of *Pfdhfr*-*Pfdhps* mutations and haplotypes in areas with different malaria transmission intensities in mainland Tanzania, 6 years after withdrawal of SP as first line drug for treatment of uncomplicated falciparum malaria.

## Methods

### Study sites

**Fig. 1:**
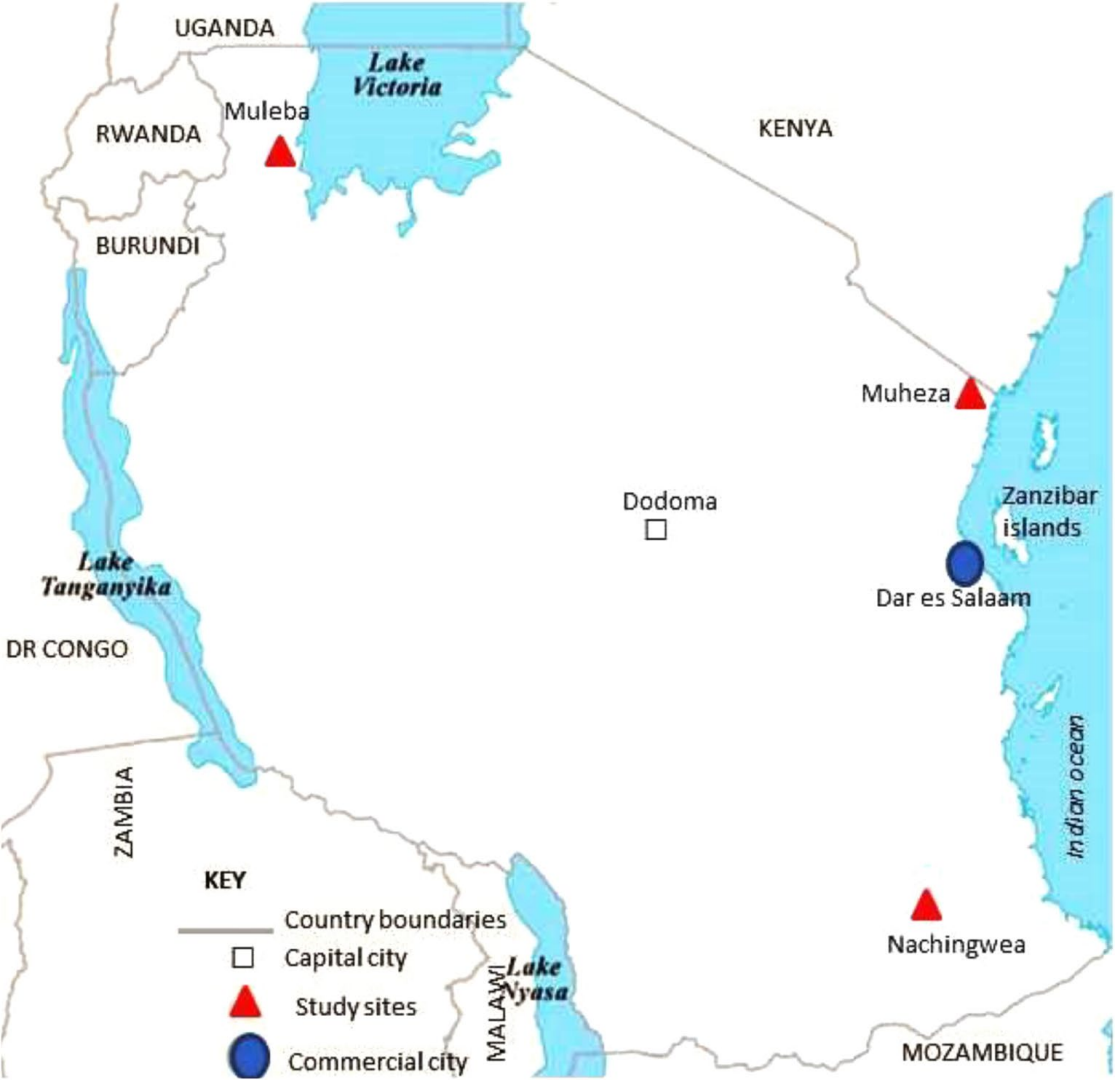
The map of Tanzania showing the study site and neighbouring countries

The samples were collected from cross-sectional surveys at three sites with heterogeneous malaria transmission intensities in Tanzania from May to August 2013. The sites included Mkuzi health Centre in Muheza District in Tanga Region, Rubya Designated District Hospital (DDH), Muleba District in Kagera Region and Nachingwea District hospital in Lindi Region. The Regions are located in north-eastern, north-western and southern zones of mainland Tanzania, respectively (Fig. 1). The study districts were selected based on evidence from the Tanzania HIV and Malaria Indicator Survey of 2012 and represent areas with low, moderate and high malaria transmission intensities [30].

### Study design and sampling methods

Cross-sectional studies were carried out in the period May–August 2013. Briefly, patients aged ≥6 months who presented at the health facilities with uncomplicated or complicated malaria were enrolled. Patients with history of fever in the past 24 h or fever at presentation (temperature ≥37.5 °C) were screened for malaria and their demographic, clinical and parasitological information were collected. Laboratory screening for malaria was done using rapid diagnostic tests (RDT) and confirmed using microscopic examination of blood smears. Venous blood samples (3–5 ml) were collected into EDTA tubes for genomic analysis of malaria parasites. Thick and thin blood smears were prepared for detection and quantification of malaria parasites. The smears were air-dried and the thin films were fixed with methanol prior to staining using 3 % Giemsa stain for 45 min. Slides were examined microscopically to count asexual or sexual parasites per 200 white blood cells (WBCs) or 500WBCs, respectively. A slide was considered negative if no parasite was detected after examining 200 fields. Parasite density was calculated by multiplying the number of asexual parasites by 40 and sexual parasites by 16 assuming that one microliter of blood contained 8000 WBCs. Haemoglobin measurement was done using a Hemocue^®^ machine (HemoCue, Ångelholm, Sweden). All patients who tested microscopically positive for malaria were treated as per national guidelines for treatment of malaria [31]. Global positioning system (GPS) coordinates were recorded using a handheld GPS device. In Muheza District, Tanga Region, samples were also collected from a clinical trial evaluating the efficacy of AL as per study protocol [32].

### DNA extraction

Blood smear microscopically positive *P. falciparum* infected blood samples were used for DNA extraction. The samples were depleted of the white blood cells (WBCs) using a CF11 cellulose columns as previously described [33]. Genomic DNA was extracted using the QIAamp DNA Blood Midi (Qiagen GmbH, Hilden, Germany) as per manufacturer’s instructions.

### Genotyping for *Pfdhps* and *Pfdhfr* mutations

The SNPs in the *Pfdhfr* and *Pfdhps* genes were detected by a combination of nested PCR and sequence specific oligonucleotide probe (SSOP)-ELISA analysis as previously described [34]. The SNPs analysed were *Pfdhfr* at codons C50R, N51I, C59R, S108N, and L164I, and *Pfdhps* at codons S436A, A437G, K540E, A581G and A613S.

### Ethical issues

The studies which contributed specimens were approved by the Tanzanian Medical Research Coordinating Committee of (MRCC) hosted by the National Institute for Medical Research, Tanzania (NIMR). Permission to conduct the study in the three districts was obtained from the relevant regional and district authorities while the heads of the health facilities gave the permission to conduct the study at the respective sites. Prior to enrolment, written informed consent was obtained from each individual or from parents/legally acceptable representative in case of children.

### Data analysis

Microsoft Access version 2007 and Excel databases were utilized for data management with double entry, validation and cleaning of the field and laboratory data. Statistical analyses were done using STATA version 11 (STATA Corp Inc., TX, USA). Characteristics of the study population for the different sites were tested by analysis of variance (ANOVA). The prevalence of the genotypes was calculated as the proportion of wild type, mixed or mutants in the total analysable samples. Mixed infections with wild-type and mutant alleles were treated as mutant while generating the haplotype prevalence. Prevalence of mutations and haplotypes between sites were compared using Chi-square or Fisher’s exact test as appropriate. Statistical analyses were performed at the 5 % significance level and 95 % Confidence Interval (CI).

## Results

A total of 264 samples from the three sites (Muheza, Muleba and Nachingwea) were included in the analysis with each site contributing 88 samples. The demographic and parasitological characteristics of the study population between the study sites were comparable (Table 1). The study populations were similar with respect to age (χ^2^ = 3.71, p = 0.16), gender (χ^2^ = 1.92, p = 0.38) and the mean haemoglobin levels (g/dL) (F = 2.5, p = 0.08). However, the geometric mean parasite density was significantly lower at Muleba compared to Muheza and Nachingwea (F = 10.9, p < 0.001), (Table 1). Fever at health facility presentation (temperature ≥ 37.5 °C) was significantly higher at Muheza (χ^2^ = 8.37, p = 0.02) compared to Muleba and Nachingwea. Only a few patients reported prior history of AL (n = 13) or SP (n = 2) usage 2 weeks prior to enrolment into the study.

**Table 1:** Demographic and parasitological characteristics of the study population in Tanzania

Parameters	Study site			P value
	Muheza (n = 88)	Muleba (n = 88)	Nachingwea (n = 88)	
Median age (years) (25–75 % IQR)	4.2 (2.1–6.3)	4.4 (2.2–13.0)	5.2 (2.9–12.6)	0.16
Sex, female n (%)	42 (47.7)	48 (54.5)	51 (58.0)	0.38
Mean haemoglobin (g/dL), (SD)^a^	10.4 (1.8)	9.9 (2.4)	9.7 (2.4)	0.08
GMPD (95 % CI)^a^	18,603 (13,280–26,060)	3700 (1899–7211)	12,968 (8066–20,848)	<***0.001***
Fever at presentation (≥37.5 °C), n (%)	65 (73.9)	59 (67.1)	47 (53.4)	***0.02***
Antimalarial treatment history, n (%)	NA	10 (11.4)	5 (6.2)	0.23

Significant values are in boldface

*IQR* inter-quartile range, *GMPD* geometric mean parasite density, asexual parasites/µL of blood

^a^One way ANOVA was used to test the differences between the study sites

### Prevalence of SNPs associated with SP resistance in *Plasmodium falciparumdhfr* and *dhps* genes

Significantly higher prevalence of *Pfdhfr* mutations at codons C50/N51**I** was detected at Muheza (100 %) and Muleba (98.8 %) compared to Nachingwea (67.5 %), (p < 0.001). At Muheza, the *Pfdhfr* mutation at codon C59**R** had reached almost saturation level (96.6 %) while other sites, Muleba (73.1 %) and Nachingwea (81.8 %), had significantly lower prevalence (p < 0.001). For codon S108**N**, the prevalence was significantly higher at Muheza (98.7 %) and Nachingwea (95.5 %) compared to Muleba (83.3 %) (p = 0.003). The *Pfdhfr* mutation at codon I164**L** was not detected at any site.

For the *Pfdhps,* significantly high prevalence of parasite carrying mutant at codons S436A/A437**G** was detected (79.8 %) at Muheza as compared to Muleba (22.2 %) while Nachingwea had none (p < 0.001). However, Nachingwea had significantly higher prevalence (61.4 %) of double mutants S436**A**/A437**G** (p < 0.001). The prevalence of *Pfdhps* K540**E** at Muheza was significantly higher (95.4 %) compared to the other sites (p < 0.001). Another *Pfdhps* mutation also associated with high level resistance at codon A581**G** was significantly higher at Muheza (p < 0.001) reaching 48.9 % in comparison to Muleba (3.9 %) and Nachingwea at which the mutations was not detected. Low prevalence of *Pfdhps* A613**S** was detected at Muleba (2/77, 2.6 %) and Nachingwea (1/82, 1.2 %).

### Prevalence of the major *Plasmodium falciparumdhfr* and *dhps* haplotypes

Similarly, the prevalence of double mutant haplotypes (C**I**C**N**I, CN**RN**I and C**IR**SI) was low except for Muleba where a significantly higher prevalence (23.6 %) of double haplotype C**I**C**N**I was reported compared to the other sites (p = 0.008). Overall, the triple *Pfdhfr* mutants (C**IRN**I) were predominant at all sites and almost near saturation at Muheza (93.3 %) but with significantly lower prevalence of and at Muleba (75 %) and Nachingwea (70.6 %), (p < 0.001).

The prevalence of wildtype *Pfdhps* haplotype (SAKAA) was significantly low at Muheza (1.3 %) compared to other sites (p = 0.003). Similarly, the three major single mutant haplotypes were also low (Table 2). The double *Pfdhps* haplotype S**GE**AA was higher at Muheza (27.2 %) and Muleba (20.8 %) compared to Nachingwea (p < 0.001) while **A**AKA**S** was low at both site (p = 0.76). About 56 % of all isolates were triple mutants with Muheza having the highest prevalence of S**GEG**A haplotype compared to **AGE**AA which was more predominant at Muleba and Nachingwea (p < 0.001), (Table 2). The *Pfdhps* quadruple haplotypes **AGEG**A were generally low and varied significantly between the sites, whereby eight isolates (10.4 %) were detected at Muheza, two at Muleba (5.6 %) and none was detected at Nachingwea (p = 0.003) (Table 2).

**Table 2:** *P. falciparum* dihydrofolate reductase (*Pfdhfr*) and dihydropteroate synthetase (*Pfdhps*) haplotypes by study sites

Gene haplotype		Prevalence of haplotypes by sites			
		Muheza (n = 77)	Muleba (n = 72)	Nachingwea (n = 85)	*P* value
*Pfdhfr*	CNCSI	0 (0)	0 (0)	3 (3.5)	0.109
	C**I**C**N**I	4 (5.2)	17 (23.6)	7 (8.4)	***0.002***
	C**IR**SI	1 (1.3)	0 (0)	0 (0)	0.642
	CN**RN**I	0 (0)	1 (1.4)	15 (17.7)	<***0.001***
	C**IRN**I	72 (93.3)	54 (75.0)	60 (70.6)	<***0.001***

		n = 77	n = 72	n = 82	
***Pfdhps***	SAKAA	1 (1.3)	11 (15.3)	11 (16.5)	***0.003***
	**A**AKAA	4 (5.2)	4 (5.6)	16 (18.8)	***0.008***
	SAKA**S**	0 (0)	1 (1.4)	0 (0)	0.308
	SA**E**AA	0 (0)	4 (5.6)	0 (0)	***0.008***
	**A**AKA**S**	0 (0)	1 (1.4)	1 (1.2)	0.76
	S**GE**AA	21 (27.2)	15 (20.8)	0 (0)	<***0.001***
	S**GEG**A	31 (40.3)	2 (2.8)	0 (0)	<***0.001***
	**AGE**AA	12 (15.6)	30 (41.7)	54 (63.5)	<0.001*
	**AGEG**A	8 (10.4)	4 (5.6)	0 (0)	***0.003***

Bold underline indicates the amino acid changes

*n* number of haplotypes excluding the mixed infections/genotypes

* Pearson Chi-square test was applied (χ2 = 39.9, p < 0.001), all others were compared using Fisher’s exact test

Upon combination of the *Pfdhfr*-*Pfdhps* haplotypes (Table 3), quadruple mutant haplotypes with single *Pfdhp*s and triple *Pfdhfr* mutation (**A**AKAA-C**IRN**I) was lowly distributed (n = 16) across all sites while the quintuple mutant haplotype, C**IRN**I-S**GE**AA was observed in only 31 isolates, at Muheza and Muleba. Sextuple C**IRN**I-S**GEG**A haplotypes (n = 32) were more predominant in Muheza whereas other sextuple haplotype combinations, (C**IRN**I-**AGE**AA (n = 69)) were mainly observed in Nachingwea. Interestingly, the emergence of the new septuple mutant haplotypes with three *Pfdhfr* and four *Pfdhps* mutant combination (C**IRN**I**-AGEG**A) was observed for the first time albeit in few samples (n = 11) and these were mainly from Muheza (n = 8) and (n = 3) were from Muleba whereas none was noted at Nachingwea. The occurrences of other *Pfdhps*-*Pfdhfr* haplotypes were generally low (Table 3).

**Table 3:** The pattern of combined *P. falciparum* *dhfr*–*dhps* haplotypes

*Pfdhps* haplotypes		*Pfdhfr* haplotypes					Total
		Wild-type	Double			Triple	
		CNCSI	C**I**C**N**I	C**IR**SI	CN**RN**I	C**IRN**I	
Wildtype	SAKAA	0	4	0	2	20	26
Single	**A**AKAA	1	3	0	4	16	24
	SA**E**AA	0	0	0	0	4	4
	SAKA**S**	0	0	0	0	1	1
Double	S**GE**AA	0	5	0	0	31	36
	**A**AKA**S**	0	0	0	0	2	2
Triple	S**GEG**A	0	1	0	0	32	33
	**AGE**AA	2	14	1	10	69	96
Quadruple	**AGEG**A	0	1	0	0	11	12
Total		3	28	1	16	186	234

The overall numbers (absolute) parasite haplotypes on the *Pfdhfr*–*Pfdhps* genes in the samples successfully analysed (n = 234). Of all the combined haplotypes, the quintuple C**IRN**I-S**GE**AA, sextuple C**IRN**I-**AGE**AA, C**IRN**I-S**GEG**A were more frequently detected

Bold underline indicates the amino acid changes, while SAKAA and CNCSI represent the wildtype haplotype for *Pfdhps* and *Pfdhfr*, respectively

## Discussion

The rapid and widespread anti-malarial drug resistance has necessitated frequent revisions of malaria treatment guidelines in *P. falciparum* malaria endemic regions. The emergence of super resistant mutations, such as the sextuple *Pfdhfr/Pfdhps* haplotypes has not only compromised malaria case management and treatment outcomes but also affected the effectiveness of the IPT strategies.

Few years after its adoption as the first-line treatment drug for uncomplicated malaria in Tanzania, several efficacy studies detected unacceptably high levels of molecular markers of parasite resistance to SP [4, 35]. Despite its replacement by ACT, SP continued to be used in the IPT strategies which is likely to provide sub-optimal effect and, therefore, monitoring the spread of resistance using molecular markers (*Pfdhfr/Pfdhps*) is still recommended [36–38]. In this study, the prevalence of A581**G** was almost 50 % in Muheza and this was significantly higher compared to Muleba (~4 %) and Nachingwea (0 %). This could confirm the earlier suggestions that this “super resistant” mutation may have originated in the north-eastern part of Tanzania and spread to other areas albeit at low prevalence [37].

A remarkable difference was observed in the prevalence of *Pfdhps* SNPs between the study sites with Muheza having the highest levels of *Pfdhps* 436/437**SG**, A581**G**, A540**E** SNPs and haplotypes compared to Muleba and Nachingwea. As expected areas with high malaria transmission intensities in Muleba and Nachingwea had higher prevalence of *Pfdhps* wild types and single mutant 436/437A**G** as compared to Muheza in north eastern where malaria endemicity has declined remarkably in recent years [39, 40]. The current WHO recommendations suggest that SP-IPTp should be discontinued if the prevalence of this double *Pfdhps* mutant, K540**E** is more than 95 % and the A581**G** is more than 10 % as it is considered to be ineffective [29]. Obviously, these criteria are still met in Muheza confirming the findings of previous studies conducted in north Eastern Tanzania [26]. In a cohort study conducted at Muheza, it was shown that IPTp–SP was associated with increased prevalence of parasites with mutations at codon A581**G** and that use of IPTp-SP conferred no benefit in improvement of pregnancy outcomes [17, 37]. Of note, this area is known to be the major focus of *Pfdhps* A581**G** mutations in East Africa which is believed to occur almost exclusively with *Pfdhfr* K540**E** leading to double mutant haplotypes [41]. The high frequency of *Pfdhps* A581**G** at alarming level in this area clearly suggests for no beneficial protective effect from the IPTp-SP [17, 18, 42]. This higher prevalence in north eastern Tanzania could be explained by the sustained drug pressure due to self-medication that could have elevated levels of SP resistance, Ringsted et al. [43] reported 76 % volume sales of SP in private drug shops in this areas. Additionally, the high prevalence levels could be maintained due to selective pressure on *Pfdhfr* and *Pfdhps* as a result of co-trimoxazole (trimethoprim-sulfamethoxazole), another antifolate, used to prevent opportunistic infections in HIV infected individuals as cross-resistance might also occur [44, 45].

The prevalence of the *Pfdhfr*-*Pfdhps* wild type haplotypes was low in all sites. The prevalence of mutant genotypes C51**I**, was at saturation in Muheza (100 %) and equally at Muleba (98.8 %) with significantly lower prevalence (67.5 %) at Nachingwea (p < 0.001). Similarly, almost complete saturation (>96 %) of other mutations in *Pfdhfr* (N51**I**, C59**R**, and S108 **N**) was observed at Muheza with marked differences between Muleba and Nachingwea (Table 2). In contrast, some previous studies have shown that other major resistance mutations in *Pfdhfr* are well established throughout the country where the *Pfdhfr* triple mutations (51**I**, 59**R** and 108**N**) were above 84 % and close to saturation in six regions of Tanzania [46]. Elsewhere, these data are in consistent with studies in West Africa where *Pfdhfr* N51**I**, N59**R**, and S108**N** have been shown to rise [19]. The prevalence of I164L mutation conferring high pyrimethamine resistance was not detected at all the three sites which is in accordance to previous reports elsewhere [47]. However, in other parts of Africa, only a few studies have reported occurrence of this high level mutation [15, 16, 48]. Of note, the *Pfdhfr* I164L mutation was first documented at low prevalence in Muheza in 1999 before the deployment of IPTp-SP [49] and later one isolate (mixed allele) was reported in Rufiji [50], but to date its presence has rarely been reported in the country.

The C**IRN**I haplotype with triple mutations was present and highly prevalent at all study sites regardless of the transmission intensity. This is in line with several studies in Tanzania and elsewhere in the SSA [51, 52]. The C**IRN**I haplotype is associated with high level resistance to pyrimethamine and increases the risk of SP resistance if it occurs concurrently with *Pfdhps* mutations [21]. The increased double mutant *Pfdhps* S**GE**AA haplotype was observed in Muheza (28 %) and Muleba (22 %), but not in Nachingwea (0 %). In Muheza, the highly resistant triple S**GEG**A *Pfdhps* haplotypes was observed, (38.7 %).

The combinations of *Pfdhfr*-*Pfdhps* were detected at higher numbers including quintuple mutant C**IRN**I-S**GE**AA, sextuple haplotype which comprise triple mutations in both genes C**IRN**I-S**GEG**A and C**IRN**I-**AGE**GA have been highly associated with sub-optimal IPTp-SP effectiveness in previous studies [17]. Interestingly, a septuple mutant haplotype C**IRN**I-**AGEG**A was observed which had not been previously reported in the study areas. From these observations, it is apparent that these mutant haplotypes associated with poor IPTp-SP are expanding in different epidemiological settings. Of worth noting, this study was not designed to correlate the clinical data with observed resistance pattern. Nonetheless previous literature showing the association of these haplotypes with clinical or treatment outcomes have been noted.

Ideally, in order to reduce the sustained drug pressure, efforts are required to limit the use of SP for IPTp purposes only through limiting over the counter SP prescriptions. Also the availability of ACT for the treatment of uncomplicated malaria and proper implementation of the national malaria treatment guidelines would also contribute in the reducing the selection pressure. However, alternative drugs for IPTp are urgently needed to replace the failing SP due to the saturation of the parasite population with *Pfdhps*-*Pfdhfr* mutations and haplotypes highly associated with SP resistance. Deployment of the sub-optimal IPTp-SP strategy is therefore unlikely to confer the anticipated effect on improving pregnancy outcomes.

## Conclusion

These results ascertain the high prevalence of *Pfdhfr* and *Pfdhps* haplotypes conferring SP resistance in areas with changing malaria epidemiology. The high prevalence of *Pfdhfr*-*Pfdhps* sextuple mutant haplotypes and the occurrence of the new septuple mutant haplotype undermine the usefulness of IPTp-SP. Thus, additional control efforts are needed to contain the spread of SP resistance and suitable alternative drugs for IPTp are urgently needed.
